# Concluding Remarks for the Special Issue on RNA Viruses and Antibody Response

**DOI:** 10.3390/v15051214

**Published:** 2023-05-22

**Authors:** Yiu-Wing KAM

**Affiliations:** Division of Natural and Applied Sciences, Duke Kunshan University, Kunshan 215316, China; yiuwing.kam@dukekunshan.edu.cn

Infectious diseases represent one of the major public health concerns on the global level. The emergence and re-emergence of different RNA viruses (influenza, SARS-CoV-1, MERS, CHIKV, Zika and SARS-CoV-2) remain a major concern for public health control worldwide [1,2]. In every disease outbreak, valuable knowledge about virus–host interactions can be learnt to better manage and control the spread of RNA virus diseases. Consequently, preventive measures such as the early detection of cases, clinical management, and the development of vaccines can be employed to reduce the socioeconomic impact of RNA-virus-mediated outbreaks. In the context of vaccine responses and immunity, the responses of neutralizing antibodies serve as predictors of protection from infection [3,4]. Understanding antibody kinetics provides important information to improve the accuracy of early detection system development. This Special Issue collects 14 original studies and 2 reviews that contribute to the overall knowledge on antibody responses triggered by RNA virus infections.

Ten original research papers are published in this Special Issue related to SARS-CoV-2, each looking at different aspects. Five studies discuss the relationship between antibody kinetic profiles and protection. These studies provide important information about whether all infected individuals will have the same course of humoral response maturation or evolve with a unique binding and neutralizing capacity which is individual specific. We might be able to use a mathematical model to predict the protection time post-infection according to antibody binding kinetics.

Currently, the detection of viral RNA by qRT-PCR remains the gold standard for the acute phase of infection. Can we use antibody detection as a diagnostic alternative? To address this, two studies in this Special Issue provide additional insights into improving the sensitivity and specificity of a diagnostic system for SARS-CoV-2 infections using either conserved full-length (N protein) or fragmented (RBD) antigens as the early serology detection system. However, more validation from new SARS-CoV-2/RNA-virus-infected cohorts will be important to ascertain the robustness of the identified “immune signature” for pathogen-specific identification. With the availability of better diagnostic alternatives, clinicians can then provide a well-informed disease intervention program for patients.

More importantly, this Special Issue contains four studies and two reviews looking at the treatment strategies for managing COVID-19 patients, particularly in preventing severe clinical outcomes post-infection. We might learn lessons from other infectious diseases (HIV and RSV) or validate different antiviral treatment options in order to improve our understanding of virus infection mechanisms and disease severity development.

RNA viruses apart from SARS-CoV-2 (influenza and CHIKV) remain a major concern for public health control globally and this Special Issue contains three studies that provide additional antibody knowledge in order to expand the development of better prophylactic and therapeutic strategies. 

We would like to thank all authors for publishing their high-quality research and reviews in this Special Issue. It has been a great pleasure working with all the talented researchers around the world. This Special Issue will serve as a platform to further improve the current knowledge of antibody responses against RNA virus infections. Hopefully, this platform will continue to facilitate the design and development of prophylactic and therapeutic strategies in preparation for future disease outbreaks.

## Data Availability

Not applicable.
